# CT pulmonary angiography findings in HIV-infected patients referred for suspected pulmonary thrombo-embolic disease

**DOI:** 10.4102/sajr.v26i1.2273

**Published:** 2022-01-31

**Authors:** Diane Wiese, Leisha Rajkumar, Susan Lucas, David Clopton, Jacob Benfield, Jason DeBerry

**Affiliations:** 1 Department of Radiology, Faculty of Medicine, Chris Hani Baragwanath Hospital, University of the Witwatersrand, Johannesburg, South Africa; 2 Department of Radiology, Faculty of Medicine, Helen Joseph Hospital, University of the Witwatersrand, Johannesburg, South Africa; 3 Department of Radiology, Faculty of Medicine, James H. Quillien VA Healthcare System, East Tennessee State University, Johnson City, Tennessee, United States of America; 4 Department of Radiology, Faculty of Medicine, Elizabeth and Claire LaPlante Foundation, West Virginia University, Morgantown, West Virginia, United States of America

**Keywords:** CTPA, HIV, pulmonary embolism, imaging findings, radiological, prevalence

## Abstract

**Background:**

South Africa bares a significant burden of HIV and imaging is commonly performed as part of the workup for respiratory distress.

**Objectives:**

The aim of this study was to document the prevalence of pulmonary thrombo-embolic disease (PTED) and other findings in HIV-infected patients referred for CT pulmonary angiography (CTPA) for suspected PTED.

**Method:**

Forty CTPA studies of documented HIV-infected individuals investigated for suspected PTED during a 1-year period were retrieved, anonymised and interpreted by three consultant radiologists. Inter-reader reliability was calculated using Free Marginal multi-rater Kappa.

**Results:**

Fourteen of the forty cases (35%) were positive for PTED. In the pulmonary embolism (PE)-positive group, 57.14% had peripheral disease and 42.86% had both peripheral and central disease. Associated findings in the PE-positive cases were pulmonary infarcts (17.5%), mosaic attenuation (17.5%) and linear atelectasis (7.5%). The most common incidental findings were solid pulmonary nodules (52.5%), non-wedge-shaped consolidation (45%), cardiomegaly (52.5%) and enlarged intra-thoracic lymph nodes (52.5%). Thirty per cent of the study population had findings related directly to the presence of PTED, whilst most cases in the study (77.5%) had pulmonary findings unrelated to PTED. In the PE-negative cases, 55% reported emergent findings that warranted immediate or urgent medical attention.

**Conclusion:**

Computed tomography pulmonary angiography imaging is critical for diagnosing PE. However, further investigation into the judicious application of CTPA in HIV-infected patients with suspected PTED is required, as CTPA findings in most of the cases in this study were unrelated to PE.

## Introduction

Multiple aetiological factors account for respiratory distress in the population affected by HIV, and many pathologies encountered share signs and symptoms of pulmonary embolism (PE). In the clinical setting of this study, CT pulmonary angiography (CTPA) is frequently requested in the workup of respiratory distress in these individuals to exclude PE. HIV has been reported to increase the risk of venous thrombo-embolism (VTE) by 2–10 fold, with a frequency reaching up to 7.6% per year.^1^ A retrospective study in Johannesburg, South Africa showed HIV to be the commonest associated risk factor for VTE.^2^

**FIGURE 1:**
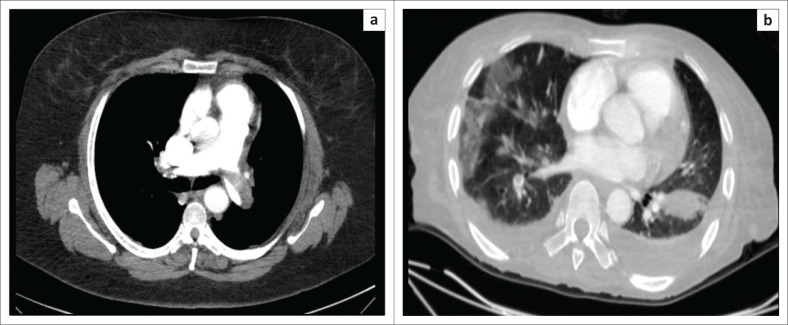
Axial CT of two different patients with pulmonary embolism. (a) Demonstrating a large saddle embolus; (b) demonstrating a filling defect in the right descending pulmonary artery complicated by bilateral pleural effusions, peripheral consolidations and a wedge-shaped pulmonary infarct on the left.

The general prevalence of PE is estimated to be 600 000 cases per year in the United States,^3^ ranging between 0.14% and 61.5% in medical patients in different African countries.^4^ Mortality is largely preventable, and thus, diagnosis of PE in hospitalised patients remains paramount to patient care and outcomes. A positive case of PE with parenchymal and pleural complications is shown in Figure 1b.

Paradoxically, studies of CTPA findings for suspected PE have also demonstrated that many alternate findings do not provide a strong rationale for its increased use.^5,6^ A study conducted in Brazil in 2016 revealed that a significant proportion of patients who had undergone CTPA for suspected PE had reported alternate findings compatible with an different diagnosis.^7^ This is a relevant consideration for resource-poor settings such as that of South Africa.

The aim of this study was to document the CT findings and the CTPA findings for the prevalence of pulmonary thrombo-embolic disease (PTED) in HIV-infected patients, given that limited data on this exist in the current literature and CTPAs are frequently ordered for the workup of respiratory distress in these individuals, which contributes further to the currently overloaded healthcare system.

## Methods

A cross-sectional, retrospective, descriptive study was conducted in the Department of Radiology at Helen Joseph Hospital, based in Johannesburg, South Africa. Convenience sampling was utilised whereby all adult HIV-infected patients who had undergone a CTPA for suspected PTED during the study period (January–December 2018) were considered for inclusion in the study. CTPA records with inaccessible digital imaging or request forms with illegible information were excluded.

Patient demographics and presenting symptoms were collected from the CTPA request forms. The HIV status was collected from request forms and/or the National Health Laboratory Service database. Patient identifiers were removed from the CTPA studies using anonymisation software. Patients in the study were scanned on a Phillips 16 slice CT scanner. 100 mL Omnipaque 350 was injected via 18 G or 20 G intravenous cannulas in the antecubital fossa using an automated pressure injector at a rate of four millilitres per second, followed immediately by a 50 mL saline bolus chaser.

The anonymised studies were read by three radiologists, each with experience exceeding four years. Readers assessed 15 criteria via questionnaire-format tick sheets for each CTPA study on Research Electronic Data Capture (REDCap). The study finding descriptors are detailed in Table 1. CT pulmonary angiography findings were classified as PE-positive by a reader based on the presence of pulmonary artery filling defects and determined overall to be PE positive if two of the three readers agreed it was positive.

**TABLE 1:** Description of terms.

Term	Description/defined as
Parenchymal	Findings limited to lung parenchyma excluding pulmonary vessels and lymph nodes
Pleural	Findings limited to visceral and parietal pleura
Cardiac	Findings limited to heart and pericardium
RV:LV	Presence of right heart strain recorded as present if the value exceeded 1^18^
MPA:AA	PAH recorded as present if the value exceeded 1:1^19^
Extra thoracic	Findings included soft tissue, visceral, bone and nodal findings above the thoracic inlet or below the diaphragm
Other intra-thoracic	Findings included abnormalities of mediastinal or hilar lymph nodes, oesophagus, bronchial arteries and anterior mediastinum
Emergent findings	Recorded as present if findings other than PE were present on the study that the reader deemed severe enough to cause respiratory distress that would warrant urgent or immediate medical attention. Examples provided in the tick sheet included pneumothorax, massive pleural effusion, extensive consolidation, cardiac failure, significant pericardial effusion, aortic dissection, haemorrhaging aneurysm, massive ascites, severe abdominal disease, tracheal or central bronchial obstruction)

RV:LV, right ventricle to left ventricle ratio; MPA:AA, ratio of main pulmonary artery diameter to ascending aorta diameter; PAH, pulmonary arterial hypertension; PE, pulmonary embolism.

Descriptive data in terms of frequencies and percentages were logged for all findings present using SAS 9.2 statistical software. Inter-rater reliability (using Randolph’s Free Marginal multi-rater Kappa) was utilised to investigate the level of agreement between the three readers.

### Ethical considerations

This study was approved by the Human Research Ethics Committee (Medical) of the University of the Witwatersrand, reference number M200256.

## Results

A total of 241 CTPAs were identified for the study period, of which 179 were excluded based on a negative or unknown HIV status, 2 were also excluded due to illegible request forms and 20 due to irretrievable digital images. The final analytical sample consisted of 40 patients. Fourteen of the 40 patients (35%) who underwent CTPA had reported PTED. The 95% confidence interval for this population proportion was between 20.63% and 51.58%.

The mean and median ages overall were 39 and 38 years, respectively, in men, and 42 and 37 years, respectively, in women. The mean and median ages of PE-positive cases were 40 and 42 years, respectively, in men, and 38 and 37 years, respectively, in women. There was a higher frequency of women compared with men, with a total of 27 women overall (67.5%). Of the 14 PE-positive cases, nine (64.3%) were women. The major presenting symptoms included shortness of breath (29.85%), chest pain (8.23%), cough (11.8%) and haemoptysis (8.8%).

Table 2 provides a detailed summary of the PE-positive CTPA findings. Twenty-seven percent had features of right heart strain, with 22.5% having a right ventricle to left ventricle (RV:LV) ratio > 1.

**TABLE 2:** CTPA findings in pulmonary embolism positive cases.

PE positive findings	Frequency	Percentage
**Pattern of involvement**		
Peripheral	8	57.14
Central and peripheral	6	42.86
**Lobe affected**		
RUL	5	12.50
RML	5	12.50
RLL	8	20.00
LUL	5	12.50
LLL	5	12.50
All (saddle embolus)	2	5.00
**Parenchymal findings**		
Pulmonary infarct	7	17.50
Mosaic attenuation	7	17.50
Linear atelectasis	3	7.00
Presence of pleural effusion	8	57.14
Presence of right heart strain (RV:LV > 1)	9	22.50
Evidence of PAH (MPA:AA > 1:1)	7	50.00

RUL, right upper lobe; RML, right middle lobe; RLL, right lower lobe; LUL, left upper lobe; LLL, left lower lobe; RV:LV, ratio of right ventricular diameter to left; PAH, pulmonary arterial hypertension; MPA:AA, ratio of main pulmonary artery diameter to ascending aorta diameter; PE, pulmonary embolism.

**FIGURE 2:**
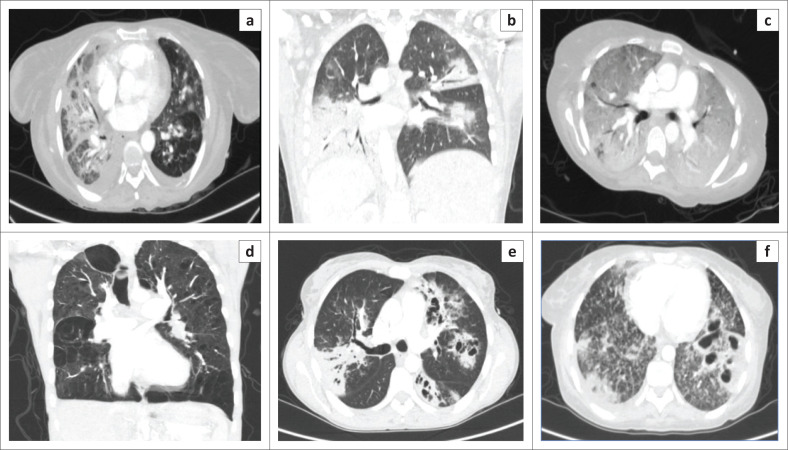
Axial (a, c, e, f) and coronal (b, d) CT slices of incidental parenchymal findings in patients without pulmonary embolism. (a–c) Extensive consolidations in three different patients, (d) diffuse emphysema, (e) cavitary multi-lobar pneumonia, and (f) left lower lobe lung cavitation with bilateral background tree-in-bud nodules in a patient with pulmonary tuberculosis.

**FIGURE 3:**
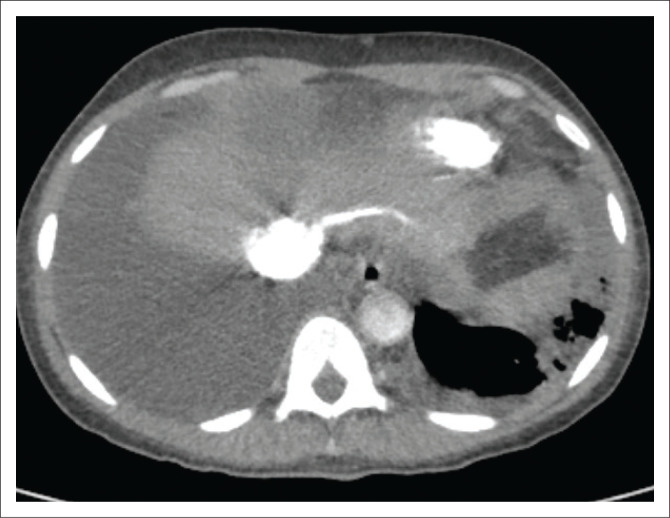
Axial CT of a patient with a large right and smaller left pleural effusion.

**FIGURE 4:**
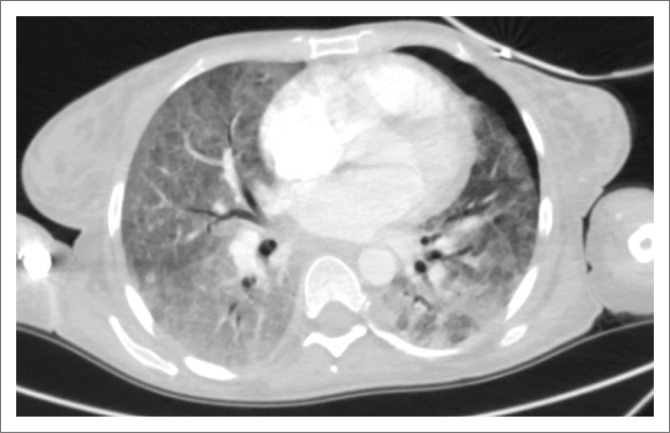
Left pneumothorax, extensive ground-glass opacities and posterior consolidations in a patient with respiratory distress.

**FIGURE 5:**
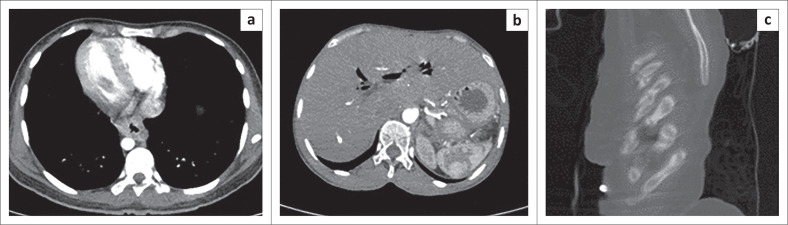
Axial (a, b) and sagittal (c) CT images of incidental extra-pulmonary findings in three pulmonary embolism-negative patients. (a) A patient with circumferential lower oesophageal wall thickening, (b) extensive pneumobilia in a post-surgical patient and (c) patient with multiple chronic rib fractures.

A complete list of the incidental findings on CTPA is summarised in Table 3 and the pulmonary findings in PE-negative cases are illustrated in Figure 2. The most common parenchymal findings in PE-negative cases were as follows: solid pulmonary nodules (52.5%), non-wedge-shaped consolidation (45%) (Figure 2a, b, c), emphysematous changes (17.5%) (Figure 2d) and lung cavities (10%) (Figure 2e, 2f). Pleural effusion was also found in 17% of PE-negative studies (Figure 3). One of the patients in the study had a pneumothorax (Figure 4). The most common incidental cardiac finding was cardiomegaly (52.5%). Various examples of extra-pulmonary incidental findings are demonstrated in Figure 5, including a case of extensive pneumobilia (Figure 5b) and a patient found to have multiple rib fractures (Figure 5c). The most common other intra-thoracic finding in PE-negative cases was enlarged mediastinal or hilar lymph nodes without significant mass effect (52.5%), whilst 7.5% had reported an oesophageal abnormality (Figure 5a); the most common extra-thoracic finding was hiatus hernia (7.5%).

**TABLE 3:** Incidental findings on CTPA for HIV-infected patients referred for suspected pulmonary thrombo-embolic disease.

Incidental findings	Frequency	Percentage
**Parenchymal**		
Non-wedge-shaped consolidation	18	45.00
Solid pulmonary nodules (> 3 mm)	21	52.50
Sub-solid pulmonary nodules (> 6 mm)	3	7.50
Ground-glass opacities	8	20.00
Emphysematous changes	7	17.50
Lung cavities	4	10.00
Lung cysts	2	5.00
Tree-in-bud	1	2.50
Pulmonary mass/es	1	2.50
**Pleural**		
Pleural effusion in the absence of PE	7	17.50
Apical pleural thickening	2	5.00
Pneumothorax	1	2.50
**Cardiac**		
Cardiomegaly	21	52.50
Pericardial effusion	1	2.50
**Other intra-thoracic**		
Adenopathy without significant mass effect	21	52.50
Adenopathy with significant mass effect	2	5.00
Oesophageal abnormality (thickening or dilatation)	3	7.50
**Extra-thoracic**		
Hiatus hernia	3	7.50
Massive ascites	2	5.00
Benign abdominal visceral lesion/s	2	5.00
Acute severe abdominal pathology†	1	2.50
Vertebral or rib fractures	2	5.00
Vertebral or rib lesions suspicious for metastases	1	2.50
Breast mass suspicious for neoplasm	1	2.50
Significant but discrete axillary or supraclavicular nodes	2	5.00
Intra-abdominal nodal masses	1	2.50

PE, pulmonary embolus.

† , demonstrated extensive pneumobilia.

The readers were 86.67% agreeable that of the PE-negative patients in the study, 55% had emergent findings that warranted immediate or urgent medical attention. Thirty percent of the study population also had associated findings related directly to the presence of PE and 77.5% had pulmonary findings unrelated to PE (Table 4). In terms of inter-rater reliability, the readers had a ‘good-to-excellent’ overall agreement, with Kappa exceeding 0.60. Specifically, for 12 out of the 15 criteria assessed, the readers had an excellent overall agreement, with Kappa exceeding 0.75.

**TABLE 4:** Frequency and percentages for the presence of emergent findings and study impression.

Emergent findings	Frequency	Percentage
**Study impression**	22	55.0
Findings related to the presence of PE	12	30.0
Pulmonary findings not related to PE	31	77.5
Cardiac findings not related to PE	4	10.0
Other significant intra-thoracic findings (mediastinal/nodal/oesophageal) not related to PE	2	5.0
Extra-thoracic significant findings	1	2.5
**Normal study**	1	2.5

Note: frequency (n), percentage (%).

PE, pulmonary embolus.

## Discussion

South Africa has one of the highest HIV burdens worldwide. Statistics in South Africa indicates the estimated total population infected with HIV in 2020 to be 13% with a steady increase of cases (from 3.8 million in 2002 to 7.8 million in 2020).^8^ One-fifth of those affected are women in their reproductive ages (15–49 years).^8^ A lowered immune system renders these individuals susceptible to a myriad of typical and atypical diseases, including bacterial pneumonias, mycobacterium tuberculosis (TB), pneumocystis pneumonia (PCP), malignancies and PE.^1^ Pathology encountered on imaging was therefore expected to be severe or atypical, and indeed, this study demonstrated many such cases. Figure 2 (c and f) illustrates diffuse ground glass opacities and cavitary disease with tree-in-bud nodules respectively – imaging findings found commonly in patients affected by atypical fungal infections such as PCP or atypical mycobacterial infections such as TB.

In terms of demographics, most patients with PE in this study were women and the average age in men and women with PE ranged from 38 to 42 years. This finding shows that PE is seen two decades earlier than in non-HIV patients where the mean age of presentation is 67–62 years.^5,6,7,9^ This is in keeping with other literature on HIV-infected patients where the mean ages for PE were younger than the general population, ranging from 40 to 45 years.^2,3,10,11,12^ Presenting symptoms in this study were typical of that described in the Prospective Investigation of Pulmonary Embolism Diagnosis (PIOPED II) trial, including shortness of breath, syncope, chest pain and haemoptysis – all of which are non-specific.^13^

The CTPA prevalence of PE in an HIV-infected study population in this study was found to be 35%. A few previous studies on the HIV prevalence in PE-positive patients have reported much lower percentages in the general population of other countries, ranging from 9.5% to 24.6%.^5,6,7,9,14^ This indicates that CTPAs are, indeed, a relevant investigation in the evaluation of respiratory distress in both HIV-infected and HIV-negative patients.

Pulmonary thrombo-embolic disease is commonly described as being ‘central’ or ‘peripheral’ in terms of location. A study conducted in China by Zhu et al. in 2012 specifically assessed the anatomical distribution of emboli on CTPAs in patients with suspected PE; they reported the central and peripheral (mixed) pattern in 55.6% and the peripheral pattern in 40.9%.^15^ The current study found more patients with a peripheral pattern (57.14%) compared to the mixed pattern (42.86%). Zhu et al also demonstrated that the right lung was more affected than the left and the lower lobes more than the upper lobes.^15^ This was echoed in a South African analysis, where lower lobes were also more commonly affected compared with upper lobes in HIV-infected patients.^11^ We reported the RLL to be most affected (20%) with a near even distribution of PE within the other lobes (Table 2), the reasons for this not being clear.

Pleural effusion was present in 57% of the PE-positive cases in this study. This is higher than the 45% in the study on HIV-infected patients by Ramlakhan et al.^11^, as well as higher than the 25.8% in the study by Sharma et al.^14^ which did not include patients’ HIV status. The most common parenchymal findings in our PE-positive group were pulmonary infarcts and mosaic attenuation (17.5% each), which is comparable with other studies carried out on both HIV-infected and -negative populations.^7,11,14^ These findings are traditionally thought to be due to PE. This study demonstrated an increased RV:LV ratio in nine of the 14 PE-positive patients (22.5%). There are no available data in the literature on the frequency of this finding in an HIV-infected study population with PE to compare with.

While our PE findings did not deviate substantially from reported literature on general populations, the incidental findings in this study were quite varied and many were significant. Most of the cases in this study (77.5%) had reported abnormal lung parenchymal findings unrelated to PE, consistent with other literature.^7,9,11,14,16^ These findings are expected in immunocompromised individuals presenting with respiratory distress.

Importantly, it was demonstrated that of the 26 PE-negative cases, 55% had emergent findings that warranted urgent medical attention (Table 4). This is significantly higher than other studies,^6,7,9^ and may, in part, be because of the injudicious use of CTPAs whereby patients with respiratory distress are referred for CTPA even when the diagnosis may not likely be that of PE. It is expected that the severity of lung changes encountered in this study is likely to have been more extensive than that found in studies on HIV-negative populations. Previous studies in general populations demonstrated lower percentages of major findings in PE-negative scans.^6,7,9^ However, the current study was limited by the smaller sample size.

The prevalence of non-wedge-shaped consolidation in this study (45%) was higher than similar studies carried out by Ferreira et al.^7^ and Sharma et al.^14^ (21.5% and 25%, respectively). These two studies examined incidental findings on CTPA for suspected PE in general populations (not HIV specific), which may account for the differences seen. The current study also documented a lower prevalence of non-wedge-shaped consolidation (45%) when compared with the 68% reported by Ramlakhan et al. which was undertaken in the Western Cape in a region known with a high TB prevalence.^11^ Only one case in this study was rated normal (2.5%) with no alternate findings, which is lower than that found in other studies.^17^

While some findings did not require immediate medical attention, they were still significant in that their presence would have likely resulted in further workup or follow-up. Obvious limitations exist in this study. Due to its retrospective nature and small sample size, this study was mainly descriptive.

## Conclusion

Pulmonary embolism is a cause of respiratory distress in HIV-infected patients and CTPA imaging is the gold standard for evaluation. However, further investigations into the prudent use and the efficacy of PE-risk determining scores should be performed regarding the request of CTPAs. HIV-infected patients presenting with symptoms of respiratory distress are not always straightforward PE cases with a multitude of other important pathologies incidentally encountered on CTPA imaging. While there is an obvious benefit to detecting a variety of emergent conditions on CTPA, further investigation into whether such conditions found in this study could be determined on other appropriate investigations, such as chest radiographs, will be of value in curbing the widespread application of CTPAs.

### Recommendation

Consideration for a future study would be to compare the chest radiograph findings in HIV-infected patients referred for suspected PE to their CTPA findings to correlate if findings on initial radiographs and CTPAs lead to markedly different diagnoses and whether this affects the treatment outcome in any significant way.
